# Cobalamin Analogues in Humans: A Study on Maternal and Cord Blood

**DOI:** 10.1371/journal.pone.0061194

**Published:** 2013-04-09

**Authors:** Tore Forsingdal Hardlei, Rima Obeid, Wolfgang Herrmann, Ebba Nexo

**Affiliations:** 1 Department of Clinical Biochemistry, Aarhus University Hospital, Aarhus, Denmark; 2 Department of Clinical Chemistry, Faculty of Medicine, University Hospital of Saarland, Homburg/Saar, Germany; Gentofte University Hospital, Denmark

## Abstract

**Background:**

Haptocorrin (HC) carries cobalamin analogues (CorA), but whether CorA are produced in the body is unknown. All cobalamins (Cbl) to the foetus are delivered by the Cbl-specific protein transcobalamin (TC), and therefore analysis of cord serum for CorA may help to clarify the origin of CorA.

**Methods:**

HC-CorA were quantified in paired samples of cord serum from newborns and serum from mothers (n = 69).

**Results:**

The CorA-concentration was higher in cord serum (median = 380, range: 41–780 pmol/L) than in serum from the mothers (median = 160, range: 64–330 pmol/L), (p<0.0001). HPLC-analysis showed CorA-peaks with retention times of 13.5, 14,5 and 16.5 min in samples from both the mother and cord serum. The peak with retention time 16.5 min constituted 24% (mother) and 45% (cord serum) of the total amount CorA, and eluted as does dicyanocobinamide.

**Conclusion:**

Our results support that CorA in the human body are derived from Cbl.

## Introduction

Vitamin B12 (Cobalamin, Cbl) is a cofactor for two enzymes in humans; methionine synthase and methyl malonyl coenzyme A mutase [1]. Cobalamin analogues (CorA) are derivatives of Cbl, unable to serve as cofactors for these enzymes.

The presence of circulatory CorA was demonstrated in the late 1970's by Allen and co-workers using measurements of plasma corrinoids with radioimmunoassay involving the two Cbl-binding proteins intrinsic factor (IF) and haptocorrin (HC) [2]. Since IF only binds Cbl and HC binds both Cbl and CorA [3], it was suggested that the discrepancy in the results obtained with the two radioimmunoassay-measurements, was caused by the presence of CorA in the extracts of plasma corrinoids. Following the initial description of CorA in humans it was discussed whether CorA were native compounds, or whether they were derived from Cbl as a result from a harsh extraction procedure [4], [5].

We have confirmed the presence of CorA on circulatory HC, while no CorA was observed on transcobalamin (TC) [6]. Our method involved immunoprecipitation of HC or TC, followed by enzymatic degradation of the Cbl binding proteins [7] and measurement of the corrinoids with a Cbl-specific and a corrinoid-specific assay [6].

The origin of CorA in the human body is unknown. One possibility is that CorA are derived from passive absorption of analogues synthesized by the microbiota of the intestine, and not endogenously created in the body from circulating Cbl in the human organism. A recent finding of large amounts of analogues in human stools [8] may support that circulating CorA originates from passive absorption from the intestine. If this hypothesis is correct, one should not expect to find CorA in the human foetus. Transport of cobalamin from the mother to the child is mediated through the TC receptor on placenta [9], [10], and no HC mediated transport have been demonstrated [11]. Thus the foetus depends solely on Cbl transported by TC, but since TC does not bind CorA [3] a presence of CorA in cord serum would strongly support that they are produced within the cells of the body from Cbl.

In the present study, we report CorA to be present in cord serum from newborns in concentrations far exceeding those in the corresponding samples from the mother and we identify the same three molecular forms of analogues in serum from mother and child. Our results strongly supports that CorA are produced within the body from circulating Cbl.

## Materials and Methods

### Study samples

Blood samples were collected from 69 at term mothers between 1 and 12 hours before they gave birth, and cord blood from the umbilical vein of their newborn babies collected immediately after expulsion of placenta. Table 1 presents the core data on the investigated population. Samples were centrifuged and serum kept at −20°C until further processed and analyzed.

**Table 1:** Main characteristics of the mothers and the newborn babies.

**Mothers (n = 69)**		
	Age in years, median (range)	30 (19–43)
	Maternal weight gain in kg, median (range)	13 (5–20)
	Caesarean sections n (%).	23 (33)
**Newborns (n = 69)**		
	Gestational age in weeks, median (range)	39 (29–42)
	Birth weight in grams, median (range)	3300 (910–4200)
	Birth length in cm, median (range)	51 (37–57)

This study cohort was recruited among deliveries at the Department of Obstetrics and Gynaecology at the University of Saarland, Germany in 2004 and has previously been characterized [12]. The original cohort consisted of 92 mother-child pairs. The present study is limited to mother-child pairs where a sufficient amount of serum was available. The local Ethics Committee at the University Hospital of Saarland, Germany approved the study, and all participants gave their informed consent to the study.

Samples of amniotic fluid were collected from 21 healthy pregnant women, 14–23 gestational weeks. These samples were leftover material from routine analysis, performed at the Department of Clinical Biochemistry, Aarhus University Hospital. According to Danish ethical rules there was no request for ethical approval.

### Investigation of cobalamin analogues on serum HC

Deglycosylation, immunoprecipitation and proteolytic degradation of serum HC or HC in amniotic fluid in order to release the protein bound corrinoids from 100 µL serum or amniotic fluid, was performed as previously described [7]. The concentration of CorA was calculated as the difference between total corrinoids (measured employing HC as the binding protein) and Cbl (measured employing TC as the binding protein) [6]. Subsequently, the resultant holoTC (as a measure for Cbl) and holoHC (as a measure for total corrinoids) was quantified with in house ELISA [13], [14].

A pool of serum from five mothers and a pool of cord serum from their babies were prepared. Corrinoids were released from HC present in 1000 µL of each of the two serum pools as described above. Before separation with reversed phase high performance liquid chromatography (HPLC), and the released corrinoids were converted into their cyano forms by exposure to potassium cyanide (KCN, Sigma-Aldrich, Denmark, 0.1 mol/L in the final solution). Standard solutions containing 200 pmol/L CNCbl or 100 pmol/L dicyanocobinamide (CN_2_Cbi, Sigma-Aldrich, Denmark) were also exposed to KCN prior to HPLC. The KCN exposed corrinoids present in the samples or standards were separated with HPLC essentially as described by Jacobsen et al [15]. In brief the corrinoids were separated at a gradient of 5–30% MeCN in 0.05 mol/L phosphoric acid pH 5 from 4 to 24 min. Post column fractions were collected for every 30 seconds between 11 min and 23 min after injection, and the eluent in the fractions was evaporated by lyophilisation (Heto-Vac, Denmark). The lyophilized post column fractions were reconstituted in 0.1 mol/L phosphate buffer containing 1 g/L bovine serum albumin (Sigma-Aldrich, Denmark) and analysed using the corrinoid- and the Cbl-specific assays and the amount of CorA was calculated as the difference between the two measures.

### Statistical analysis

All statistical analysis (Standard linear regression and Students *t*-tests and paired *t*-tests) were performed with the software program GraphPad Prism version 4.00 (GraphPad Software, San Diego, California, USA). Original data, as part of this study, can be requested from the corresponding author.

## Results

**Figure 1:**
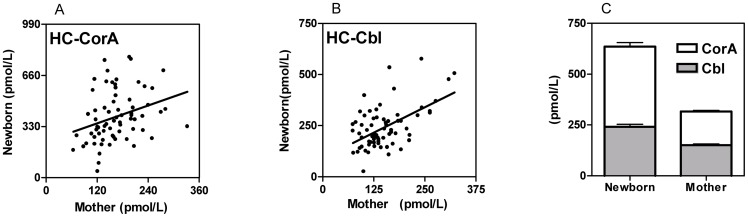
Cobalamin analogues (CorA) and cobalamins (Cbl) on haptocorrin (HC) in maternal and cord sera from 69 mother-baby pairs. (A) Results for mother and newborn showed a weak correlation for HC-CorA (p = 0.01), and (B) a strong correlation for HC-Cbl (p<0.0001). (C) The absolute and relative (HC-CorA/HC-corrinoids)) amounts (mean and SEM) of HC-CorA was higher in serum from the newborns (390±20 pmol/L, (0.62±0.016)) than in serum from the mothers (170±6 pmol/L, (0.53±0.014)), p<0.0001 (paired or unpaired t-test) for both parameters.

In this study we examined Cbl and CorA attached to HC in serum from term mothers and cord serum from their newborn babies. Table 2 presents the measurements of HC bound Cbl (HC-Cbl) and HC bound CorA (HC-CorA). Serum from the newborn babies contained a significantly higher concentration of HC-Cbl and HC-CorA than did their mothers (paired *t*-test, p<0.0001 for both measurements). In addition, the fraction of corrinoids on HC accounted for by CorA, was higher in the newborn babies, than in the mothers (paired *t*-test, p<0.0001). While the concentration of HC-Cbl showed a significant direct correlation between values in cord serum and that in maternal serum (linear regression, r^2^ = 0.29, p<0.0001) the contents of HC-CorA showed only a weak correlation (linear regression, r^2^ = 0.093, p = 0.01) (Figure 1 A and B). Serum from the newborn babies contained 2.5 (range 1.0–8.3) fold the amount of CorA and 1.6 (range 0.71–3.9) fold the amount of Cbl present in serum from the mothers. No difference was observed between results obtained for babies born by Caesarean (n = 21) or vaginal (n = 48) delivery (data not shown).

**Table 2:** Measurements of cobalamin (HC-Cbl) and cobalamin analogues (HC-CorA) attached to serum HC.

	Median	Range
**Newborns (n = 69)**		
HC-Cbl	220 pmol/L	25–580 pmol/L
HC-CorA	380 pmol/L	41–780 pmol/L
Fraction accounted for by CorA	0.64	0.14–0.89
**Mothers (n = 69)**		
HC-Cbl	140 pmol/L	74–320 pmol/L
HC-CorA	160 pmol/L	64–330 pmol/L
Fraction accounted for by CorA	0.54	0.23–0.77

**Figure 2:**
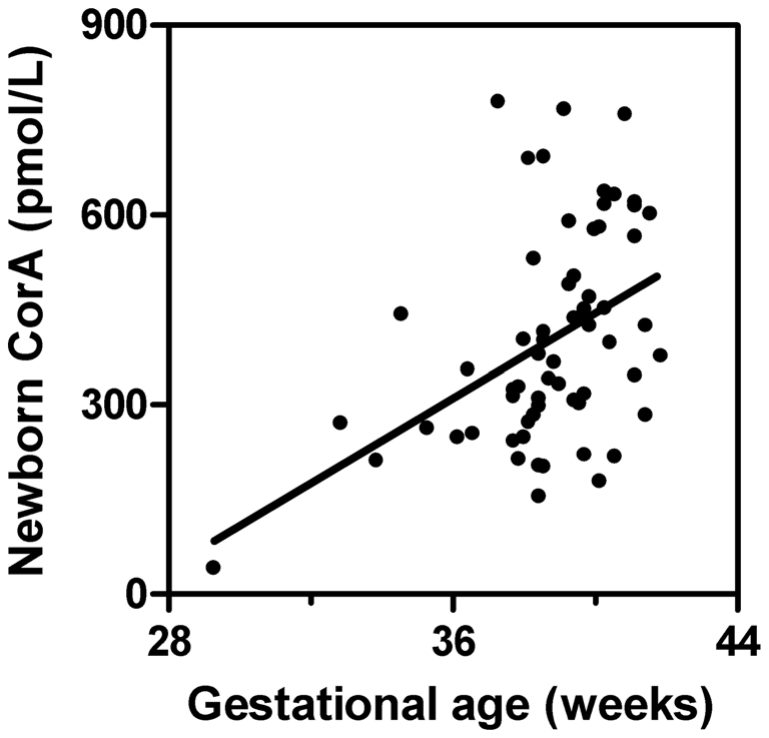
HC-CorA in cord blood in relation to gestational age of the newborns. The concentration of CorA in cord serum correlated to the gestational age (r^2^ = 0.19, p = 0.0004) of the newborns (n = 63).

The babies showed a limited variation in gestational age. Nevertheless, the concentrations of HC-CorA in cord serum showed a weak linear correlation with the gestational age (linear regression, r^2^ = 0.19, p = 0.0004). Because gestational age was unknown on 6 of the newborns in the cohort, only 63 datasets was included in this calculation (Figure 2).

The amount of HC-Cbl present in amniotic fluid at gestational age (range and (median) 14–23 (20) weeks (n = 21) showed a range (median) of 43–740 (170) pmol/L. The corresponding data for HC-CorA was 2–218 (147) pmol/L and for the fraction HC-CorA/(HC-Cbl+HC-CorA) the results was 0.01–0.59 (0.36). We found no significant correlation between gestational week and the concentration of HC-Cbl or HC-CorA in amniotic fluid.

**Figure 3:**
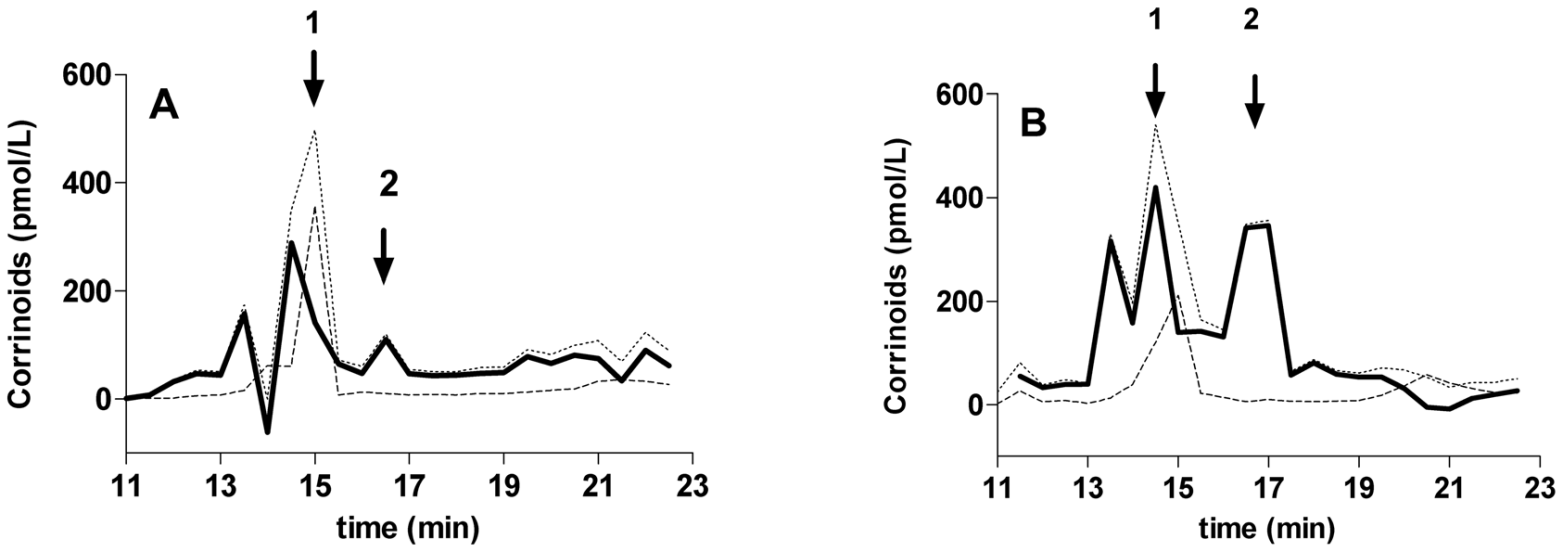
HPLC separation of cobalamin analogues (CorA). Corrinoids were enzymatically released from immunoprecipitated HC derived from a pool of serum from mothers (A) and cord serum (B), exposed to KCN and separated by reversed-phase HPLC at a gradient of 5–30% MeCN in 0.05 mol/L phosphoric acid pH 5 from 4 to 24 min. Corrinoids in the post column fractions were quantified with the corrinoid-assay (……) and the Cbl-assay (----). The elution pattern for CorA was calculated as the difference between the two (**——**). Three distinct CorA peaks were identified in serum from both mother and newborn eluting at 13.5, 14.5 and 16.5 min. Retention times for CNCbl (15 min) (1) and CN_2_Cbi (16.5 min) (2) are indicated.

Enzymatically released corrinoids from HC in pooled serum from mothers and in pooled serum from their babies were separated with HPLC after converting of all the corrinoids forms into cyano forms, and post column fractions were analyzed. As expected, cobalamin eluted as cyanocobalamin upon treatment with KCN, giving rise to a peak with a retention time of 15 min in both chromatograms (Figure 3). This peak was recognized with both the Cbl-assay and the corrinoid-assays. Three additional peaks were identified in both pools, and recognized only with the corrinoid assay as CorA. These peaks showed retention times of 13.5 min, 14.5 min and 16.5 min. The latter peak has a retention time similar to CN_2_Cbi.

## Discussion

This study demonstrates the presence of relatively high amounts of CorA in cord serum from newborn babies. We also detected both Cbl and CorA in amniotic fluid. Because of that it may be questioned whether foetal CorA is derived from the amniotic fluid, through foetal swallowing and intestinal uptake. We find this very unlikely since the intestinal IF-Cubam mediated pathway is specific for Cbl, and do not promote the uptake of CorA [for review, see 16]. Contrary, CorA in amniotic fluid is likely to be derived from the foetus. From studies in adults it is well known, that HC mediates the hepatic uptake of both Cbl and CorA. Both compounds are excreted in the bile, and while Cbl is recycled CorA is excreted in the faeces [for review, see 17].

Foetal Cbl stems from placental uptake of TC-bound Cbl, while no transport of HC-bound corrinoids across the placenta seems to occur [11]. TC recognizes only Cbl and thus our data supports that CorA is produced within the foetus. At birth, the ratio between mean values of CorA for cord and maternal sera is 2.5 and thus exceeds that of HC-Cbl (ratio 1.6).

The fact that Cbl in the foetus is derived from the mother, explains the correlation between maternal and cord sera HC-Cbl concentrations. The correlation is driven by two other correlations. The Cbl-status of the newborn mirrors that of the mother, and the HC-Cbl status is related to the Cbl status [18]. The weak correlation between concentrations of CorA in maternal and cord sera supports that the relation is driven solely by the level of Cbl rather than by a transport of CorA from mother and child – or from child to mother. The correlation between the concentrations of CorA in cord serum and gestational age of the new born babies (Figure 2) suggests that CorA accumulate during foetal life.

HPLC studies of corrinoids from cord serum, revealed that a substantial amount (45%) eluted with the same retention time as did KCN exposed Cbi. Even though more CorA was released from cord serum than from the maternal serum, the molecular forms of CorA appears to be the same, and comparable to peaks observed in KCN exposed corrinoids present on serum HC from healthy donors [6].

Our results have several implications. First, the relatively high occurrence of a compound eluting as does Cbi, suggests an ordered cleavage of the phosphate group linking the corrin ring to its lower ribose-benzimidazole ligand. Second, the observation that the same types of CorA are present in the foetus and in the mother suggest that also in the mothers the CorA is derived from a degradation of Cbl rather than from intestinal absorption of CorA. A high amount of CorA has recently been reported to occur in stools [8], an amount high enough to suggest that passive absorption of CorA could occur in the large bowel. However, studies of KCN exposed CorA from human faeces showed that only about 2% of the corrinoids produced by intestinal microbiota was Cbi [8], thereby making it unlikely that the CorA recovered from serum is derived directly by passive absorption of Cbi.

In conclusion our data suggests that CorA is produced in the human body from Cbl. The exact nature of the corrinoids and also the mechanism by which they are formed remains to be clarified.

## Acknowledgments

The technical assistance of Anna Lisa Christensen and Jette Fisker Petersen is highly appreciated.
